# One Health in hospitals: how understanding the dynamics of people, animals, and the hospital built-environment can be used to better inform interventions for antimicrobial-resistant gram-positive infections

**DOI:** 10.1186/s13756-020-00737-2

**Published:** 2020-06-01

**Authors:** Kathryn R. Dalton, Clare Rock, Karen C. Carroll, Meghan F. Davis

**Affiliations:** 1 Department of Environmental Health and Engineering, Johns Hopkins Bloomberg School of Public Health, 615 N Wolfe St, W7034G JHSPH EHE, Baltimore, MD 21205 USA; 2 Division of Medical Microbiology, Department of Pathology, Johns Hopkins University School of Medicine, Baltimore, MD USA; 3 Department of Molecular and Comparative Pathobiology, Johns Hopkins University School of Medicine, Baltimore, MD USA

**Keywords:** Infection prevention, Infection control, Hospital-associated infections, Hospital environment, HAI interventions, One Health

## Abstract

Despite improvements in hospital infection prevention and control, healthcare associated infections (HAIs) remain a challenge with significant patient morbidity, mortality, and cost for the healthcare system. In this review, we use a One Health framework (human, animal, and environmental health) to explain the epidemiology, demonstrate key knowledge gaps in infection prevention policy, and explore improvements to control Gram-positive pathogens in the healthcare environment. We discuss patient and healthcare worker interactions with the hospital environment that can lead to transmission of the most common Gram-positive hospital pathogens – methicillin-resistant *Staphylococcus aureus*, *Clostridioides* (*Clostridium) difficile,* and vancomycin-resistant *Enterococcus* – and detail interventions that target these two One Health domains. We discuss the role of animals in the healthcare settings, knowledge gaps regarding their role in pathogen transmission, and the absence of infection risk mitigation strategies targeting animals. We advocate for novel infection prevention and control programs, founded on the pillars of One Health, to reduce Gram-positive hospital-associated pathogen transmission.

## Introduction

One Health approaches are based on the belief that we cannot truly understand human, animal, and environmental health by addressing each in isolation. In order to address complex public health challenges, we must understand the interconnectedness of these domains with a holistic methodology. Similar to other systems-thinking models, One Health focuses equally or more on the relationships between the factors in the system, rather than on the individual-level factors themselves.

The One Health paradigm has origins in the recognition that diseases often emerge from interactions of humans and animals, termed initially as “one medicine”, and incorporated preventative and public health. It has since grown to include environmental science and eco-health to encompass the shared environment role [1]. The combined assessment of health risks across the three domains; humans, animals, and the environment; involves design and implementation of intervention strategies that address all three sectors with a goal to produce assimilated knowledge. The One Health concept has been successfully applied to fields such as emerging zoonotic disease outbreak investigation and biosecurity risk across humans and animals [1, 2].

**Fig. 1:**
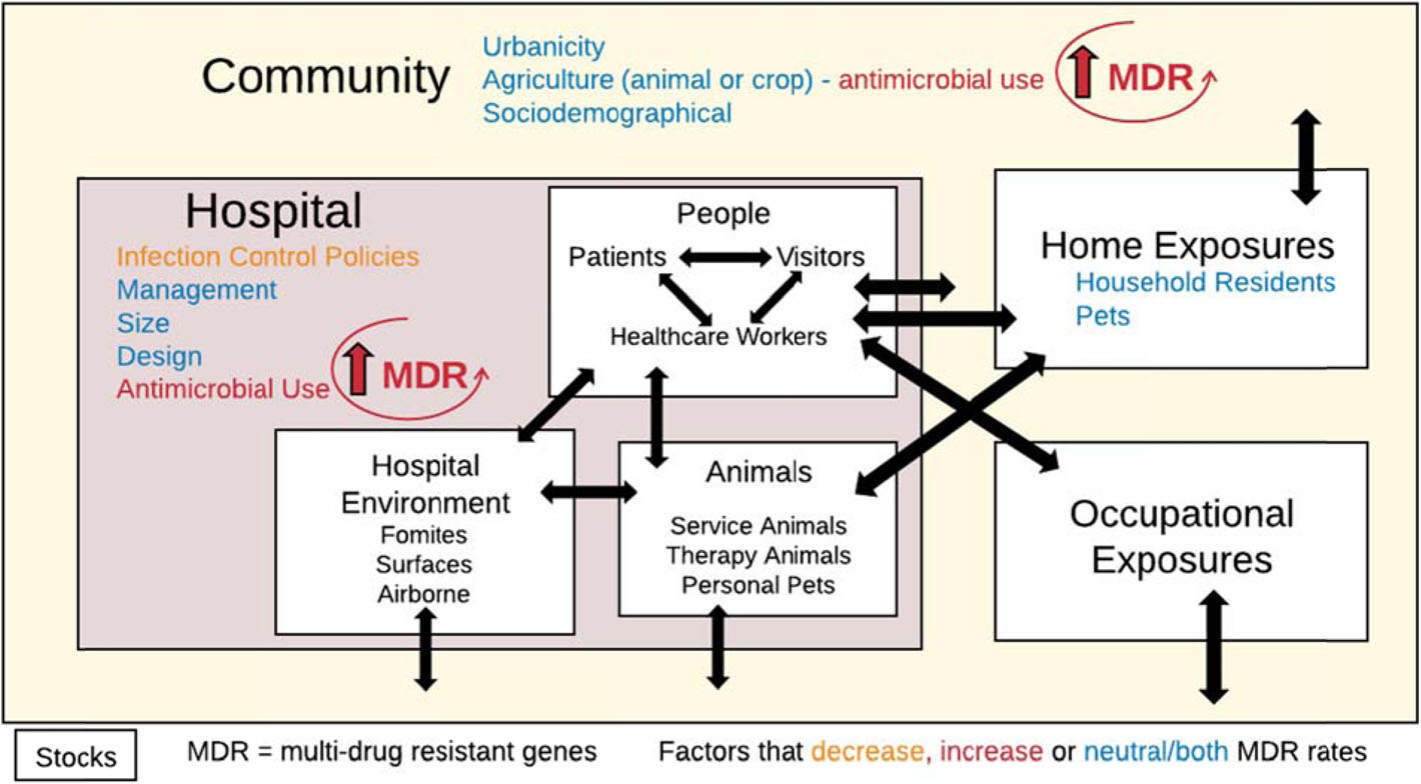
Interaction of Humans, Animals, Hospital Environment, and the Community in Hospital-Associated Pathogen Transmission

But how does One Health impact our healthcare system? Hospitals serve as an incubator that incorporates dynamic microbial inputs from the community from both people and animals, as illustrated in Fig. 1. Antimicrobial use exerts selective pressure on these incoming microbial ecosystems, shifting to a higher prevalence of resistant organisms. Microbial ecosystems are defined for this paper as the composition, and the networks, of the entire microorganism population within a single niche or site. Individuals in the hospital (both patients and employees) may become colonized with hospital-associated multidrug-resistant organisms (MDRO) and then are discharged back to the community, creating a cyclic feedback loop [3–5]. Finally, MDRO acquisition and infection is more likely diagnosed in the hospital setting, resulting in the hospital serving as both a surveillance point and multiplier for resistant organisms and infections, which underscores the need to describe community and hospital-based risk factors that influence the hospital environment.

**Fig. 2:**
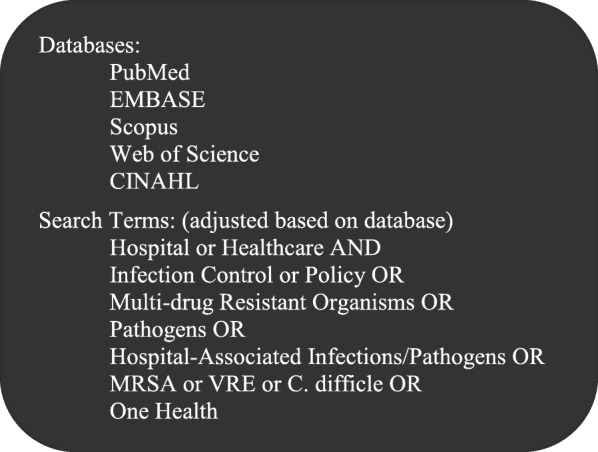
Literature Search Methodology

The application of One Health principles to hospital infection prevention and control has not been described previously. In the clinical setting, One Health can provide practical ways to incorporate environmental and animal contact considerations into patient care. While the concept has been endorsed by major medical and public health organizations, studies of physicians reveal limited awareness to the environmental health aspects of medical problems in the patient care settings, as well as low awareness levels about prevention or treatment of zoonotic diseases from animals [6, 7]. Therefore, the purpose of this review is to use a One Health lens to describe the relationship between the hospital environment and patient care specifically for Gram-positive hospital-associated pathogens, and to identify how animals fit into this relationship (Fig. 1). A broad literature search was conducted to identify information relevant to the scope of this work, see Fig. 2. Articles published prior to June 2019 were considered for review.

### Hospital-Associated Gram-Positive Pathogens

Healthcare-associated infections (HAI) are an increasingly prevalent threat in the Unites States healthcare system. The Centers for Disease Control and Prevention’s National Healthcare Surveillance Network (CDC-NHSN), a US surveillance system, estimates that about one in 31 hospitalized patients acquires an HAI [8]. This review focuses on Gram-positive bacterial pathogens, a significant cause of HAIs, which may survive longer on dry surfaces than Gram-negative bacteria [9, 10]. Methicillin-resistant *Staphylococcus aureus* (MRSA) was the first pathogen where spread through the hospital environment was documented, though targeted hospital efforts are contributing to its decline in the past decade [11, 12]. It is relevant to One Health, as some MRSA strains and other multidrug-resistant staphylococci are associated with animals, livestock in particular [13, 14].

The second most common hospital associated Gram-positive pathogen is *Clostridioides difficile* (genus recently reclassified from *Clostridium* [15]). Rates of resistance and transmission depends on strain, with higher rates seen in the PCR ribotype 027 and 078 epidemic strains, and documented resistance to quinolones, clindamycin, rifamycins, erythromycin, chloramphenicol, tetracycline and even imipenem [16]. It is included in this review because it is the most common hospital-acquired infection pathogen (~ 500,000 infections annually with up to 30,000 deaths in the US) and antibiotic prescribing for other infections (such as MRSA) can be a risk factor for *C. difficile* infection, conversely treatment with the recommended vancomycin protocol has been shown to lead to our third Gram-positive pathogen of concern [16].

The third Gram-positive pathogen we cover in this review, of increasing concern as a hospital-associated pathogen, is vancomycin-resistant *Enterococcus* (VRE). While not credited with the same degree of pathogenicity as MRSA or *C. difficile*, VRE causes infections in vulnerable patients, including outbreaks that are difficult to control due to its resistance to routine cleaning. All three important Gram-positive pathogens are able to survive in the environment for days to months and have low infectious doses—as low as 5 spores (*C. difficile*) or 4 CFUs (MRSA)— where inadequate environmental approaches can pose an ongoing risk of transmission to hospital patients [17].

## The Hospital Environment

### The hospital built-environment

Critical to a One Health approach is the role of the environment, including the unique characteristics of the built environment. The built environment is defined as the infrastructure created by people for spaces where they live and work, with consideration for how physical properties of these buildings influence health [18]. The hospital environment can facilitate transmission of pathogens responsible for HAIs. The inanimate environment can be a MDRO reservoir [19, 20], with environmental contamination responsible for approximately 10–30% of patient MDRO acquisitions [21].

Table 1 summarizes select key studies on the role of the hospital environment in MDRO and other pathogen transmission. Contamination of high-touch surfaces with MDROs such as methicillin-resistant *Staphylococcus aureus* [39, 40], vancomycin-resistant *Enterococcus* [25, 39], and *Clostridioides difficile* [34, 41] for prolonged time periods has been well documented, and thus can serve as a potential reservoir for onward infections to patients and healthcare workers. Multiple studies have shown that there is higher HAI risk for patients who are in rooms that were previously occupied by an HAI-positive patient, even after routine cleaning and disinfection [29–31].

**Table 1:** Key Studies that Examine the Role of Environment in Patient Infectious Disease Outcomes

Relation	Organism	Comments	Reference
**Increased Acquisition**			
ENV - > Patient	MRSA	Outbreak of MRSA in hospital that lasted 2 years was found in hospital dust with the same genotype.	[22]
ENV - > Patient	Not specific	Patients assigned to shared bay rooms had a 21% greater relative risk of a central line infection (*p* = 0.005), compared with patients assigned to private rooms. At the hospital level, a 10% increase in private rooms was associated with an 8.6% decrease in central line infections (*p* < 0.001), regardless of individual patients’ room assignment.	[23]
ENV - > Patient	MRSA	Three of 26 patients who acquired MRSA while in the intensive care unit acquired MRSA from the environment, strains from the patients and their immediate environment were indistinguishable	[24]
ENV- > HCW	VRE	Contact with contaminated surfaces in the rooms of colonized patients results in transfer of VRE to gloved hands, despite cleaning with disinfectants	[25]
ENV - > HCW	*C. difficile*	Increasing levels of environmental contamination was positively associated with increasing amounts of *C. difficile* on the hands of healthcare workers, particularly for environmental sites that patients touch	[26]
Patient - > ENV	*C. difficile*	Surfaces in rooms exposed to a *C. difficile* patient had significantly increased odds of being contaminated with *C. difficile*, compared to surfaces in unexposed patient rooms	[27]
Patient - > HCW	MRSA	Two-thirds of staff enter a room containing an MRSA patient will acquire the patient’s strain on gloved hands or apron, even without touching patient directly (40%)	[28]
Patient - > Patient	MRSA, VRE	Admission to a room previously occupied by an MRSA-positive patient or a VRE-positive patient significantly increased the odds of acquisition for MRSA and VRE.	[29]
Patient - > Patient	*C. difficile*	Prior room occupant with CDI was a positive risk factor for new patient CDI acquisition, hazard ratio 2.35 *p* = .01	[30]
Patient - > Patient	Several (MRSA, *C. difficile*, VRE)	Pooled acquisition odds ratio of 2.14 (95% confidence interval (CI), 1.65e2.77) for several bacteria from prior occupants, Gram positive 1.89 (95% CI: 1.62–2.21)	[31]
Patient - > ENV - > HCW	MRSA	In the colonized patient’s room, HCW exposure occurred more predominantly through the indirect (patient to surfaces to HCW) mode compared to the direct (patient to HCW) mode.	[32]
**Cleaning/Removal Reduces Human Acquisition**			
ENV - > Patient	MRSA	Enhanced cleaning during an outbreak decreased the number of new affected patients, stopped outbreak, and saved an estimated ﻿£28,000.	[22]
ENV - > Patient	General	Lower infection rates associated with routine disinfection of surfaces (mainly floors)	[33]
ENV - > Patient	*C. difficile*	Daily disinfectant high touch surfaces and dedicated cleaning staff reduced CDI positive cultures by 60%	[34]
ENV - > Patient	*C. difficile*	Hydrogen peroxide vapor decontamination reduced CDI rate by 37%	[35]
ENV - > Patient	VRE	Hydrogen peroxide vapor reduced VRE by 80%	[36]
ENV - > Patient	MRSA	Reduction in acquired MRSA infections with enhanced targeted cleaning compared to routine cleaning, despite higher MRSA patient-days and bed occupancy rates during enhanced cleaning periods (*P* = 0.032: 95% CI 7.7, 92.3%). Genotyping identified indistinguishable strains from both hand-touch sites and patients	[37]
ENV - > HCW	VRE	Decreasing VRE contamination of environmental surfaces decreases hand colonization of VRE and VRE acquisition rates	[38]

*MRSA* methicillin-resistant *Staphylococcus aureus*, *CDI C. difficile* infection, *VRE* vancomycin-resistant *Enterococcus*, *ENV* hospital environment, *HCW* healthcare worker

Aspects of the hospital’s built-environment and design, including different surface materials, can influence microbial transmission. Plipat et al. showed that MRSA may more easily and in higher burden contaminate porous surfaces, but when those contaminated porous surfaces are touched by patients or healthcare workers they are less likely to transfer MRSA compared with non-porous surfaces [32]. Another example of hospital design is private versus open shared rooms. A review of over 1 million inpatient records from 335 US hospitals found a 10% increase in private rooms was associated with an 8.6% overall decrease in hospital-associated catheter infections [23, 42]. Other hospital level risk factors for patient HAI acquisition include larger hospital size and higher patient density and clustering [43–48]. Hospitals that are highly connected to one another through a shared health-care system or through a referral system have more patient MRSA bacteremia incidence rates (partial correlation coefficient *r*  =  0.33 (0.28 to 0.38)) [49, 50]. Another key hospital design consideration is hospital-acquired pathogen strains may enter into the community through improperly treated hospital wastewater effluent, including MRSA and VRE [51], although discussion of this topic is beyond the scope of this paper.

#### Hospital fomites

Inanimate objects within the hospital can frequently become contaminated with pathogens and serve as sources for contamination and potential colonization for individuals who come in contact with them. These important fomites can travel between hospital rooms and patients, serving as a mechanical vector in pathogen spread. Nearly any item in contact with skin can serve as a fomite in pathogen transmission, from wearables like white coats and ties to pens, medical devices, and mobile telephones. Hospital objects have been extensively sampled for pathogen carriage and colonization, with prevalence rates as high as 55% for stethoscopes, 52% for neckties, and 50% for rings [52]. Concise reviews of the major reservoirs have been published previously by the Centers for Disease Control and Prevention’s “Guidelines for Environmental Infection Control in Health-Care Facilities” [53] and in the International Society for Infectious Disease’s “A Guide to Infection Control in the Hospital” [54]. Other possible dissemination routes for pathogens, including. *S. aureus* and *C. difficile*, is airborne dispersion [55–57], promoting spread among the hospital environment and individuals.

#### The hospital microbial ecosystem

However, human exposure to resistant pathogens occurs in the context of microbial ecosystems, and the hospital built environment can be a source for a number of other microorganisms that are less often pathogenic but can serve as potential reservoirs of resistant genes. A hospital microbiome can harbor a diverse set of antimicrobial resistance genes that are extremely relevant to human health, and these ultimately could be reflected in HAI rates. For example, there is evidence for frequent horizontal transfer of the mobile genetic element Staphylococcal Cassette Chromosome *mec* (SCC*mec*) gene, which encodes for methicillin resistance, between *S. aureus* and coagulase-negative *Staphylococcus* [58]*.* Coagulase-negative staphylococci are not traditionally regarded as pathogenic, but share the same ecological niche in the human anterior nares, leading to the opportunity for horizontal gene transfer [58]. Understanding other potential sources of antimicrobial-resistant genes is fundamentally important in combating and understanding MDRO epidemiology. Bacterial diversity also varies among different hospital areas – it has been shown that the halls, living rooms, patient rooms, and rest rooms exhibit more diverse bacterial compositions than that of the isolated ICU [59]. Different ICU management practices, including more rigorous sanitation protocols, could exert selective pressure and foster survival of microorganisms that express genes for resistance to common disinfectants and antimicrobial agents [60].

Within the hospital built environment, humans are a predominant source of colonizing microbes. Researchers found that bacteria in a patient room resembled the skin microbiota of the patient occupying the room and became more similar throughout the patient’s stay [61]. Additionally, they reported that patients acquire microorganisms that were present in the room before patient admission, indicating transfer both ways between patients and the hospital environment of all microorganisms—not just pathogens [61]. This means that patients and hospital workers likely alter the hospital’s microbial composition in the specific areas they occupy, resulting in unique micro-environments within the larger hospital. While this currently is an understudied research area, a better understanding of how microorganisms colonize, persist, and change in the hospital environment has the potential to elucidate major infection sources beyond attempts to focus on specific pathogens, and provide key insights into human health.

## Human Factors

### Patient characteristics

Human factors are critical when assessing One Health in hospitals in the context of HAI transmission. According to some estimates, 5–10% of patients will develop an infection while in the hospital [62]. Multiple studies have shown that around 10% of patients who enter hospitals are asymptomatically colonized with at least one type of MDRO, emphasizing the substantial influx of MDRO from community settings into the hospital [63]. A mathematical model of hospital pathogen spread showed that increasing the patient MDRO prevalence at admission to 12%, or doubling the average length of hospital stay, almost tripled the predicted overall prevalence of MDRO-colonized patients within the hospital [64]. Established factors associated with increased risk of nosocomial infection include prolonged antimicrobial therapy, comorbidity with chronic health conditions, compromised immune function, and close proximity to other patients infected or colonized with an MDRO [4]. Higher patient density, from both higher influx or longer length of patient stay, can increase direct contact rates between patients which could increase the probability of direct transmission of MDRO. In addition, because patients shed bacteria into their local environments, patient density can also increase contamination of the environment and environmental fomites, thereby increasing the indirect transmission of MDRO [4]. An increasing reservoir of MDRO through increases in patient admission or length of stay is important to address when assessing the efficacy of infection control interventions. If the reservoir of MDRO increases, then the benefits of preventive strategies may be minimized. Studies have shown higher prevalence of HAIs in hospitals within more densely-packed urban centers, hospitals in lower socioeconomic neighborhoods, and hospitals in communities where the majority of residents are racial and ethnic minorities, independent of hospital risk factors [65–68].

Patients are often prescribed antibiotics as part of their hospital care, occasionally untargeted and unnecessarily, as published reports have estimated that 23–46% of antibiotic prescriptions are inappropriate [69–74]. This widespread antibiotic use places selective pressure on bacterial ecosystems, enhancing survival of bacteria with resistant genes. Such pressure has been shown to affect horizontal gene transfer rates between bacterial species [75]. While most hospitals have antimicrobial stewardship programs that implement guidelines for judicious antimicrobial use, often antibiotic use is critical to patient care. This often creates an environment that is conducive to the persistence of resistant pathogens. It has been well-documented that selective pressure from antimicrobials increases the MDRO bacterial load colonizing patients, and that the higher bacterial load leads to greater patient skin and hospital environmental contamination [76]. Conversely, the absence of selective pressure from antimicrobials results in lower MDRO bacterial loads and leads to a lower likelihood of skin and environmental contamination [77]. The genes from resistant bacteria can spread to the hospital environment and other individuals in the hospital, then spread to the greater community. Cycling of such strains from the community can be another route for re-entry into the hospital.

#### Role of healthcare workers

A primary transmission route of hospital-associated pathogens for patients is through contaminated healthcare workers (HCW). Thirty to 40 % of HAIs may be spread by contaminated healthcare worker hands—hands that were contaminated either from direct contact with infected or colonized patients, or from their environment [41]. Even without direct patient contact, healthcare workers can serve as vectors and spread pathogens between environmental surfaces throughout the hospital [78]. A meta-analysis and systematic review calculated that the pooled MRSA prevalence among HCW in non-outbreak settings was 4.4% (95% CI, 3.98–4.88%), with nursing staff at increased risk for MRSA carriage; nursing staff had an odds ratio for MRSA colonization of 2.58 (95% CI 1.83–3.66) when compared with other healthcare staff [3]. While contamination is typically found on HCW hands, other wearable fomites, such as stethoscopes, digital devices, white coats, and neckties, can commonly be contaminated with bacterial pathogens including MRSA [52, 79]. Studies have concluded that pathogen transmission from colonized patients to HCW gowns and gloves is substantial, particularly for those whose job duties involve high contact activities [80].

In addition to the potential role HCWs play as vectors, increasing the risk of colonization and infection to patients, there is also the occupational safety concern for infection to the workers themselves. Hospital employees serve a critical function in society; a decreased labor force due to illness from infectious disease can have detrimental economic consequences [81]. In a 10-year study across Dutch hospitals, there were 17 reported MRSA outbreaks: 13 outbreaks involved HCWs, and in 8 cases HCW acquired MRSA infections despite following the current safety precautions, showing that HCWs are at risk as much or more so than the patients during these outbreak situations [82]. Other occupational safety conditions, such as elevated stress, poor supervision and leadership, and weak communication networks, can increase nosocomial pathogen spread [83]. Increased patient density and overcrowding combined with understaffing may lead to failure of MRSA control programs through decreased HCW hand-hygiene compliance, increased patient and staff movement between hospital wards, and overburdening of screening and isolation facilities [84]. Subsequently, high MRSA incidence leads to increased inpatient length of stay, which can exacerbate conditions of overcrowding and foster a feedback loop that perpetuates HAIs [84]. Similar to patients, HCWs could play a more active role in community transmission due to the greater frequency of hospital exposure, although this hypothesis has not been tested.

In addition to patients and HCWs, a hospital receives many daily visitors who contribute to the microbial composition of the hospital environment. It is estimated that the prevalence of pathogen colonization, including Community-Associated MRSA (CA-MRSA), in healthy asymptomatic individuals ranges from 0.2 to 7.4% [85–87]. These studies showing higher prevalence rates in community visitors compared to common patient or HCW carriage rates may be partly due to success of infection prevention and control policies such as environmental cleaning and hand hygiene compliance in HCW and patients [88]. Of note, individuals who visit the hospitals may be there for contractual service, such as for deliveries. Because these individuals are not considered employees of the hospital, they may not be as well trained on infection control measures nor may be subject to the infection control policies and practices that are job requirements of hospital-employed HCW. This is another understudied area in existing literature.

## Animals in the Hospital

The final aspect of One Health that has received less attention in the context of hospital-associated pathogen control is the roles of animals. Table 2 summarizes selected studies that describe the relationship between humans and animals in the spread of infectious diseases. Animals are potential sources of pathogens, including ones commonly considered nosocomial, which can spread to humans. It has been documented in multiple studies that MRSA strains found in companion animals such as dogs and cats are identical to epidemic strains found in human hospitals [90, 93, 109]. There are many ways that animals, and their corresponding and unique microbial ecosystems, can positively and negatively enhance transmission of infectious pathogens. Exposure to animals, from pets in the home to farm animal exposure, can increase an individual’s overall microbial diversity, which can then be protective against colonization of opportunistic pathogens [115–117]. This balance of being both a supply and deterrent of human pathogen colonization is the reason why animals are so essential to examine in any context, including the hospital environment. Our understanding regarding direction of transmission, colonization persistence, animal-human transmission rate, animal carriage and inter-species transmission risk factors, and the significance of companion animals as reservoirs for human pathogens are all incomplete.

**Table 2:** Selected Studies on Potential Transmission of Pathogens between Humans and Animals in Various Settings

Organism	Comments	Reference
**Ecological**		
MRSA	MRSA strains found in companion animals such as dogs and cats are identical to epidemic strains found in human hospitals	[89]
MRSA	Resistance patterns and genetic make-up of MRSA isolates from dogs and cats are generally indistinguishable from the most prevalent hospital-associated MRSA strains in the human population	[90]
MRSA	Increase in companion animal MRSA, including MDRO, same clonal lines as CA&HA-MRSA	[91]
MRSA	Phylogenomic analyses showed that companion animal isolates were interspersed throughout the epidemic MRSA pandemic clade and clustered with human isolates from the United Kingdom suggesting a human source for isolates infecting companion animals	[92]
**Pet Ownership**		
MRSA	﻿Transmission of MRSA occurs between humans and companion animals and vice versa	[93]
MRSA	Identification of indistinguishable MRSA isolates in both pets and humans in contact with them	[94]
MRSA	MRSA was found in pets from MRSA-positive owners in 4/49 (8.2%) vs. none of the pets of the 50 uninfected human controls. ¾ of these pairs had concordant PFGE pattern	[95]
MRSA	MRSA-infected animal was initially identified, at least one MRSA-colonized person was identified in over one-quarter (6/22; 27.3%) of the study households. By contrast, only one of the 8 (12.5%) study households of MRSA-infected humans contained a MRSA- colonized pet	[96]
*Enterococcus*	76% of the isolates from companion dogs had belonged to hospital-adapted clonal complex, screening of 18 healthy humans living in contact with 13 of the dogs under study resulted in the identification of a single, intermittent carrier. This person carried one of the sequence types recovered from his dog	[97]
MRSA	Identical strains from both pets and their owners were identified. Typical livestock-associated *S. aureus* lineages were observed in humans and/or companion animals and hospital and/or community-acquired *S. aureus* lineages were detected among pets.	[98]
*C. difficile*	PFGE patterns of some dog and human *C. difficile* isolates were over 90% similar	[99]
**Livestock**		
MRSA	373 (9.7%) patients coming from a high-density farming area were positive for MRSA, 292 (78%) had livestock-associated MRSA strains and 81 (22%) non-LA-MRSA strains	[100]
MRSA	Patients exposed to pigs or veal calves were shown to have 3 times higher incidence of MRSA colonization	[101]
MRSA	MRSA carriage in HCWs in contact with livestock is 10-fold higher than in other HCWs	[102]
**Hospital**		
MRSA	Dog was implicated as a reservoir for the re-infection of two nurses after their treatment to eliminate carriage of MRSA	[103]
MRSA	Cat residing in a geriatric rehabilitation ward was implicated as the source of MRSA for nurses and patients	[104]
MRSA, *C. difficile*	Zoonotic agents were isolated from 80 out of 102 (80%) dogs who visit hospitals, primary pathogen was *Clostridium* [sic] *difficile*, which was isolated from 58 (58%) fecal specimens, Seventy-one percent (41/58) of these isolates were toxigenic	[105]
MRSA	Acquisition of MRSA by a pet therapy dog that had visited an elderly care ward in a healthcare facility	[106]
MRSA, *C. difficile*	Rates of acquisition of MRSA and *C. difficile* were 4.7 and 2.4 times as high, respectively, among dogs that visited human health-care facilities, C.diff 4% was toxigenic, MRSA hospital origin clone	[107]
*C. difficile*	Canine fecal isolate from healthy dog who visits hospitals was indistinguishable from the major strain implicated in outbreaks of highly virulent CDAD, which were occurring at increased frequency in the facility around the time the dog’s fecal specimen was collected	[108]
**Veterinary Hospitals**		
MRSA	Cluster of five canine postoperative wound cases infected with MRSA were found to be associated with asymptomatic carriage of MRSA in one of the attending veterinary surgeons. The human and canine isolates were corresponded to the predominant epidemic strain prevalent in hospitals at this time	[109]
MRSA	MRSA was isolated from 16% (14/88) of household contacts or veterinary personnel and in all 6 identified cases at least one human isolate identical to the initial animal isolate was found.	[110]
MRSA	Comparison of genetic markers shows that identical or very similar strains disseminate among animals and veterinary personnel. Companion animals harbor PVL-positive clones - Twenty-six pets and five veterinary personnel carried PVL-positive S. aureus	[111]
MR Staph	Risk factors for nasal colonization by MRS in healthy humans: (i) being a veterinary professional (veterinarian and veterinary nurse) (*p* < 0.0001, odds ratio [OR] = 6.369, 95% confidence interval [CI, 2.683–15.122]), or have contacted with one MRSA- or MRSP-positive animal (*p* = 0.0361, OR = 2.742, 95% CI [1.067–7.045]	[112]
MRSA	One veterinary nurse, who carried Panton Valentine leucocidin-positive ST338 MRSA, also owned a ST749 MRSP-positive dog	[113]
MRSA	MRSA was isolated from 14 staff (17.9%), four dogs (9%), and three environmental sites (10%), which all had the same PFGE pattern.	[114]

*MRSA* methicillin-resistant *Staphylococcus aureus*, *C. difficile Clostridioides difficile*, *HCW* healthcare worker

### Pets in the home

There have been numerous examples of microbial sharing between people and their pets in the household, and pet ownership is a risk factor to acquire, maintain and spread potential pathogenic bacteria. For example, Ferriera et al. found, in 49 MRSA-infected outpatients households, 4 cases of MRSA colonization in companion animals (8.2%), 3 of which shared PFGE patterns from their owners, and no MRSA positive pets in the negative human control households [95]. That study also found a human who was infected with MRSA resided with a dog colonized with methicillin-resistant *Staphylococcus pseudintermedius*, a common veterinary pathogen in companion animals that occupies a similar niche as *S. aureus* and causes similar disease conditions in animals. It was hypothesized that SCC*mec* could have transferred between the related bacteria [95]. Another study found similar findings; one of the 8 (12.5%) study households of MRSA-infected humans contained a MRSA-positive pet; conversely they also evaluated human colonization in homes with a MRSA-carrier pet and determined that over 25% (6/22; 27.3%) owners were MRSA-positive [96]. This higher association of pathogen carriage for humans and pets in the same households, and the identification of indistinguishable MRSA isolates in both pets and humans in contact with them, strongly suggests interspecies transmission but it does not indicate the direction of transmission. However, given the preponderance of common human MRSA clones in household pets, it is possible that animals become contaminated through contact with colonized or infected humans and that they in turn serve as a source of re-infection or re-colonization [92]. Given that pets may clear carriage or contamination with removal from infected owners, veterinary guidance recommends contact isolation for household pets in the case of recurrent MRSA infection among humans in the household [118].

### Pets in the hospital

Animals can contribute to the hospital microbial ecosystems by directly entering the hospital. A patient may require a service animal, which according to the Americans with Disability Act, have the legal right to enter the hospital. Therapy animals are employed in many healthcare settings and may visit multiple patients and visitors during their time in the hospital. Therapy animals are particularly important because they can visit multiple patients, multiple wards, and even multiple hospitals all within the same day [119, 120], indicating their potential as an effective mechanical vector in the spread of pathogens. Finally, some hospitals allow for periodic or routine visits from patients’ personal pet(s) during inpatient care; in a survey of 337 SHEA member hospitals, 121 (36%) healthcare facilities allowed personal pet visitations, of which 7 (5.8%) did not have formal guidelines in place [121]. In addition, resident animals in healthcare facilities have been known to be vectors of hospital-pathogens, such as the case reports of a cat residing in a geriatric rehabilitation ward, or a nurse’s visiting pet dog that were implicated as the sources of MRSA outbreaks [103, 104]. Since then, few studies have evaluated zoonotic disease carriage of therapy animals living in or entering the hospital. Lefebvre et al. found that 80 out of 102 (80%) asymptomatic therapy dogs who visited hospitals had a zoonotic pathogen positive fecal sample. The primary pathogen was *C. difficile*, which was isolated from 58 (58%) fecal specimens; 71% (41/58) of these were toxigenic and many were genotypically indistinguishable from the major strain implicated in ongoing outbreaks of highly virulent human *C. difficile* acute disease [105, 108]. The group also identified that acquisition rates of MRSA and *C. difficile* were 4.7 and 2.4 times higher, respectively among therapy animals compared to household dogs, indicating their increased contact with hospitals could increase exposures to HAIs, similar to human risk factors [107]. Service animals, therapy animals, and personal pets will have different exposures, and thus have different microbial compositions. Just as patients can bring microbes into the hospital from the community, animals can also serve as a vector between the hospital and community, and their unique microbial ecosystems could impact this vector function.

### Food animals

In addition to household pets, food animals, such as beef and dairy cows, poultry, and swine, each have unique microbial compositions and can influence pathogens circulating in the community and the hospital. Although the use of healthcare-prescribed antimicrobials in humans is an important risk factor in MDRO colonization in the population and environment, the use of antimicrobials in food animal production also contributes—at times substantially—to the reservoir of resistance [122]. Medically-important antimicrobial drugs may be used in food animal production, as well as companion animal practice, contributing to selection for and emergence of pathogens resistant to specific drugs, including those of critical importance to human medicine. Food animal uses of antimicrobial drugs can influence the hospital environment indirectly via MDRO-contaminated meat or other food products, indirectly via exposure of community members who live in proximity to agricultural production, and directly via animal contact. For example, in a study matching MRSA-colonized incoming patient cases to non-colonized control patients, cases had over 4 times higher odds of living near swine-rearing facilities [123]. Another study found that MRSA carriage in HCWs in contact with livestock is 10-fold higher than in other HCWs [102]. Similarly, patients exposed to pigs or veal calves in Denmark were shown to have three times higher incidence of MRSA colonization [101]. Finally, in another study, 373 (9.7%) patients coming from a high-density farming region were MRSA-positive, which is similar to what is found in other non-rural settings, but 292 (78%) had livestock-associated MRSA strains rather than HA- or general CA- strains [100]. For more detail, other reviews have been published which discuss the role of livestock and food agriculture operations in the spread of community pathogens [124–127].

## Interventions to Reduce Exposure

**Fig. 3:**
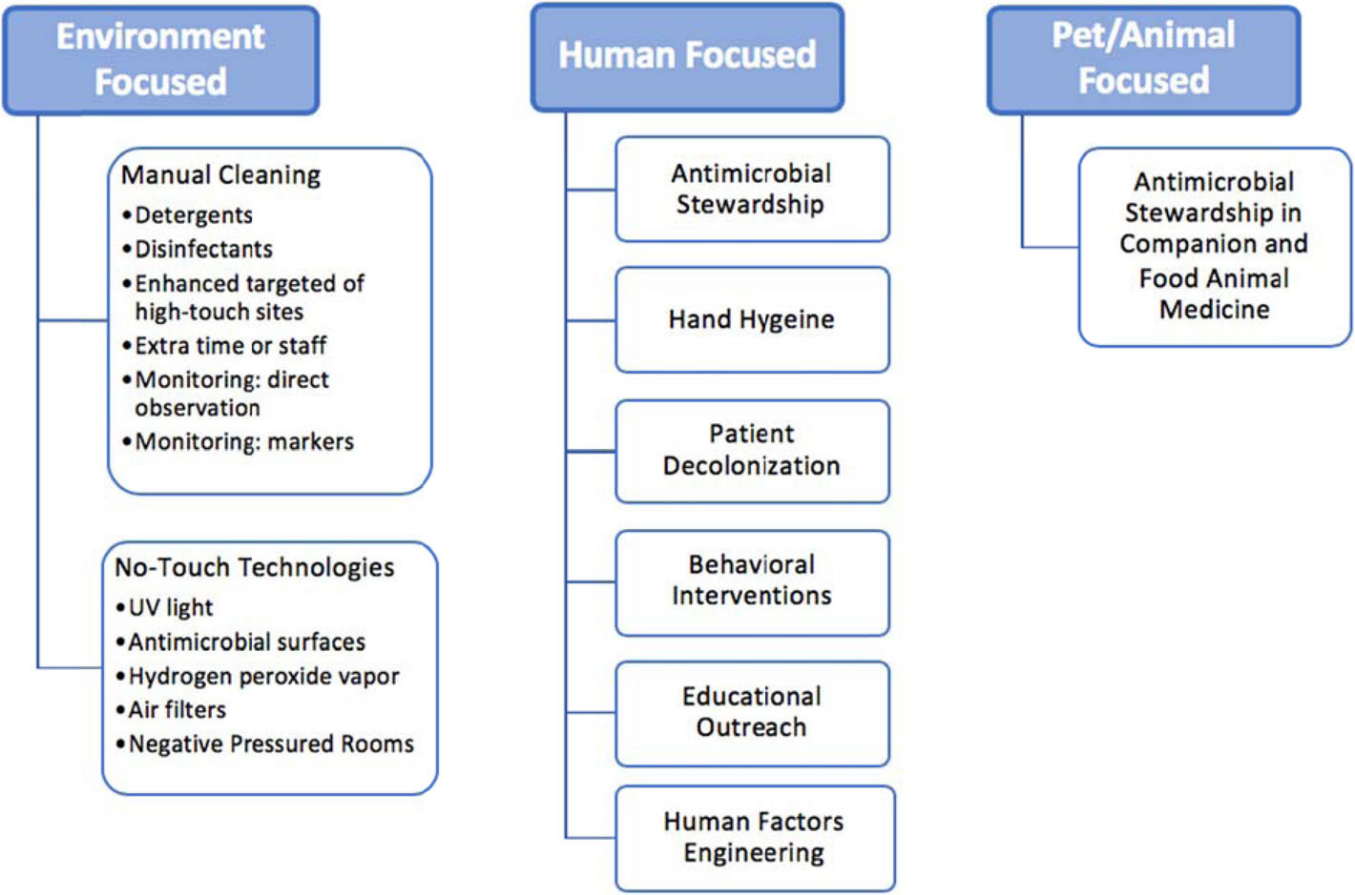
Examples of Infection Prevention and Control Strategies within the One Health Domains

**Fig. 4:**
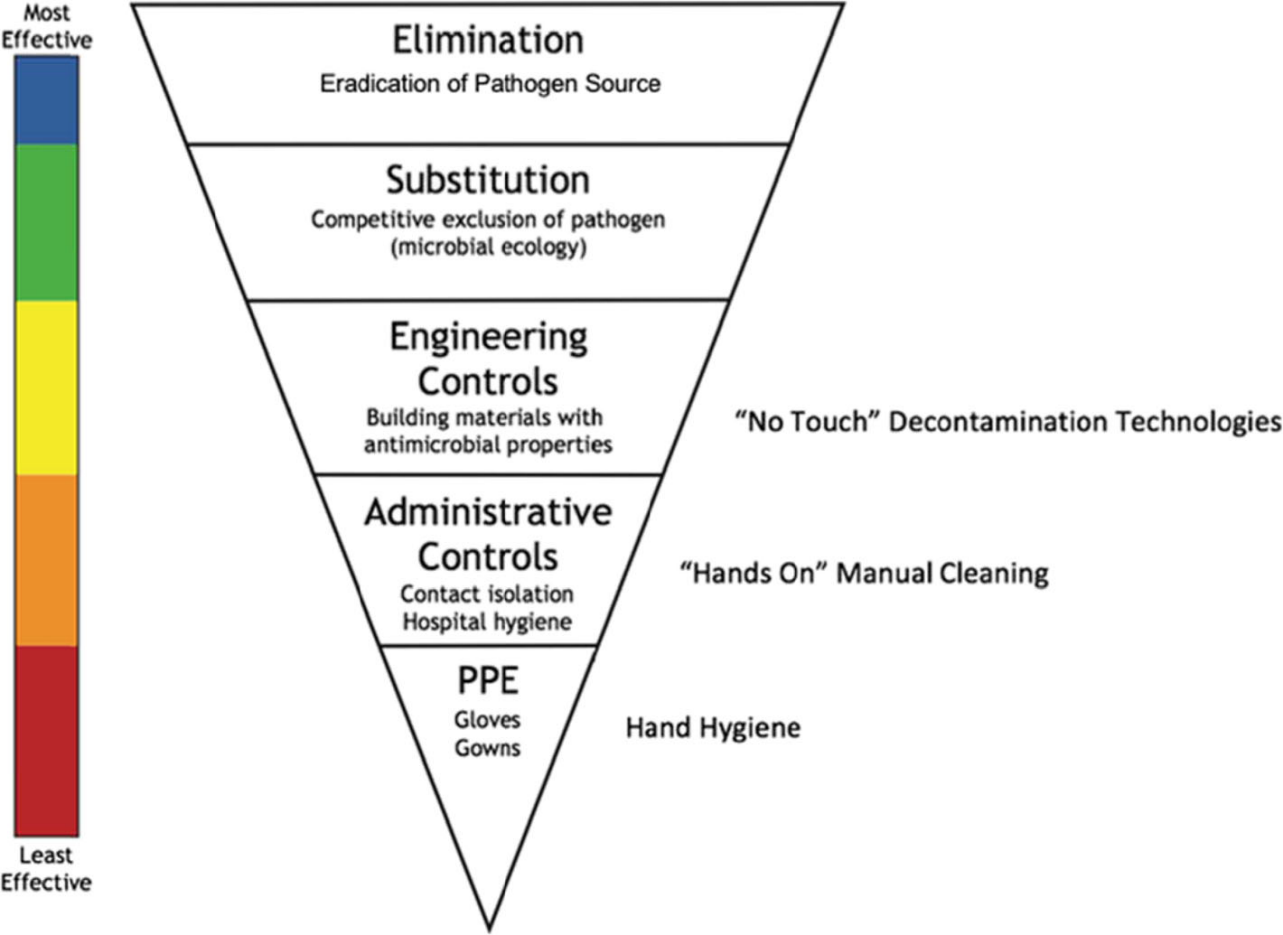
How Infectious Disease Intervention Strategies relate to the Hierarchy of Controls. Legend: Adapted from the National Institute for Occupational Safety and Health (NIOSH, www.cdc.gov/niosh/topics/hierarchy); PPE: personal protective Equipment

The challenge of complex microbial and pathogen inputs from community sources to the hospital environment—and the pathogen dynamics among individuals who are treated, visit, and work within in this setting—requires an integrative perspective to design interventions to reduce the risk of human exposure, colonization, and infection. Therefore, focusing on individuals by themselves or a single type of MDRO may provide incomplete answers. Microbes, including pathogens, circulate between the hospital environment and the larger community, with individuals and animals serving as mechanical vectors. Most interventions are designed to target only one sector, but multimodal strategies may be more successful to break this cyclic feedback loop. Addressing the hospital environment and animal sectors can reduce human exposure of microbes and pathogens, and human-focused interventions can reduce colonization risk. We will discuss interventions within each One Health domains, as shown in Fig. 3, and their effectiveness to address community-level factors and patient infectious outcomes. For this review, effective interventions are defined as those which reduce or nullify exposure or colonization risk yet are feasible to implement in a clinical setting, using the CDC NIOSH’s (National Institute of Occupational Safety and Health) Hierarchy of Controls as a strategy for ranking the effectiveness of interventions, as shown in Fig. 4, where those grouped in the top of the graphic are potentially more effective and protective than those at the bottom. For MDRO control, elimination or substitution, the most effective forms of prevention against hazards would equate to elimination of the source of pathogen, such as creating policies that control animals into the hospital, thus limiting the risk of “sick” animals potentially carrying zoonotic MDRO into the hospital. Engineering and administrative controls, such as changing hospital design or altering hospital safety culture, can be effective but do not nullify the exposure hazard. Personal protective equipment (PPE), such as gloves and gowns, are the most simplistic form of control measures, as they rely heavily on human motivation and are prone to human error, so should not be the sole means of infection control, as evident by multiple studies showing variance in PPE compliance [128–131].

### Hospital interventions

Interventions targeted at the environmental sector have been shown to have downstream benefits on the microbial carriage and colonization of humans [33, 34, 132]. In the literature, interventions targeting the hospital environment are centered on “hands-on” manual cleaning/disinfection protocols and “no-touch” decolonization technologies and isolation through facility or administrative design or through other engineering controls. Cleaning with detergents has been shown to reduce MRSA levels that exist in the healthcare environment; however, detergents can be inferior at killing microbes compared to disinfectants, and cleaning products can become contaminated, furthering the spread of pathogens in the hospital [133, 134]. Disinfectants, while shown to decrease bacterial burden on a surface, can also release toxic fumes and can cause allergic and hypersensitivity reactions in HCW, which may limit the feasibility of increased use [135, 136]. Cleaning activities are behaviors and therefore may be more effective when monitored, either by direct observation, which is relatively easy and inexpensive but susceptible to human error, or with fluorescent markers, which offer an objective assessment of residual contamination after cleaning [137, 138]. A number of studies suggest that targeted cleaning focused on highly-touched common fomites is more effective than general cleaning, not only in efficacy of actual decontamination but also in effectiveness, since this intervention is feasible to implement frequently [32, 40]. However, there are limitations to typical cleaning procedures. Microbial properties of organisms, including biofilm development, can make them more resistant to detergents, and even common disinfectants [139, 140]. A randomized controlled study that evaluated increasing daily cleaning frequency and targeted disinfection showed only modest decreases in patient VRE infections (relative risk 0.63, 95% CI 0.41–0.97, *p* = 0.034), and no changes in the incidence of *S. aureus* bacteremia (RR 0.82, 0.60–1.12, *p* = 0.22) or *C. difficile* infection (RR 1.07, 0.88–1.30, *p* = 0.47) [141]. This indicates that cleaning itself is imperfect, possibly prone to human error. This is best captured in a natural experimental study by Vietri et al., which found that a hospital move and adoption of radical new cleaning procedures did not result in a statistical decrease for MRSA colonization rates in patients and HCW [142].

“No-touch” technologies include decolonization strategies that may be less prone to human error. These include UV irradiation, which has been shown to be effective as a terminal disinfectant process after initial cleaning preformed to remove debris, but was seen to vary substantially based on location in a room relative to the UV device [143, 144]. In addition to patient isolation rooms, aspects of the hospital built-environment design can contribute to infection control. Chiefly, certain surface materials have antimicrobial properties, although these have been found to be variable [145]. Kim et al. found that the use of titanium dioxide-based photocatalyst antimicrobial coating on common environmental touch surfaces significantly decreased MRSA acquisition rates in hospital patients (hazard ratio of contracting hospital-acquired pneumonia during the intervention period compared to baseline period: 0.46; 95% confidence interval 0.23–0.94; *p* = 0.03) [146]. Other no-touch environmental interventions include aerosolized hydrogen peroxide vapor, HEPA-filtration systems, and negative-pressure rooms, which minimize aerosolized microbes and have been shown to be effective against MRSA and *C. difficile* [147–149]. If utilized, it is recommended these strategies are used as adjuncts to best cleaning and disinfection practices. Unanswered questions remain – when to use disinfectants versus detergents, when to focus on no-touch decontamination processes versus hands-on manual cleaning, and how best to monitor interventions and measure their effectiveness.

### Human interventions

Human-centered interventions reported in the literature have focused primarily on hygiene: patient decolonization, HCW hand hygiene, and wearable fomites decontamination. A meta-analysis evaluating patient washing with chlorhexidine washcloths and wipes in a hospital setting identified a total HAI rate reduction (odds ratio (OR): 0.74; 95% confidence interval (CI): 0.60–0.90; *p* = 0.002), although studies had moderate heterogeneity (I (2) = 36%) [150]. This effect was more evident in the Gram-positive subgroup (OR: 0.55; 95% CI: 0.31–0.99; *p* = 0.05) [150]. HCW hand-hygiene campaigns are a major component of multi-faceted infection control interventions, and a separate meta-analysis showed it had the strongest effectiveness for reducing nosocomial infection rates (median effect 49%, effect range 12.7–100%) compared to other interventions [151]. However, hand-hygiene campaigns alone had a modest effect size. Other facets of a bundled infection prevention and control bundle include antibiotic stewardship, another key pillar of human-centered infection control [151]. Part of this may be due to the imprecise relationship between HCW’s risk perceptions and how these perceptions affect their use of risk-mitigating strategies. In fact, demographic, individual and organizational factors, including management structures, were found to influence risk perceptions and HCW’s adoption of infection control strategies [151]. Studies that have evaluated reasons for this disparity and ways to improve behavior to prompt adequate hand-hygiene protocol addressed determinants of knowledge, awareness, action control, and facilitation of behavior. Fewer studies addressed social influence, attitude, self-efficacy, and intention, but the study authors found that addressing combinations of different determinants showed better results [152]. Increased surveillance and targeted interventions against those colonized have been shown to be effective in some circumstances [153, 154]. However such strategies have not been sufficient to control outbreaks in other situations [155–157] and are generally not recommended due to the high resource burden [158]. Contact precautions and isolation of patients known to be colonized with target pathogens has also been shown to be effective, although this is not a substitute for proper hygiene protocols [159].

A recent advance in human-centered interventions is the adoption of human factors engineering, which is a discipline that studies the capabilities and limitations of humans and the design of devices and systems for improved performance. In the context of hospital infection control, this deals with designing spaces and opportunities for individuals to avoid exposure and colonization to pathogens, a form of administrative control. This has the potential to identify major underlying causes and contributors to a problem. It goes beyond education and training, which are often the focus of infection prevention interventions, to modify an individual’s context so that default decisions align with healthy and desired actions. It utilizes environmental design, such as handwashing or antiseptic alcohol stations at the exits of patient rooms and one-way human traffic flows, in a way that minimizes exposure to healthcare workers and other patients to effect downstream reductions in the contamination of other hospital surfaces and individuals [42]. This relies heavily on proper leadership for both implementation and oversight. Human factors engineering systems models with audit and feedback, when applied, can increase effective room cleaning and disinfection, decreasing bacterial bioburden in the patient room [160, 161]. An example of this is the addition goal setting and HCW engagement, resulting in a hospital safety climate, was associated with improved compliance (pooled odds ratio 1.35, 95% confidence interval 1.04 to 1.76; I (2)=81%) compared to the standard of training and education, observation and feedback, and reminders [162].

### Animal interventions

Just as animals have not been extensively examined in their role as vectors of pathogens and other microbes, there are also few studies on interventions in animals in either a hospital or community setting. Just as intervention programs focus on hand hygiene protocols in HCW because of their role as vectors of hospital-associated pathogen transmission between patients and the hospital environment, therapy and service animals may also fill a similar niche, but infection control programs that target animals in healthcare settings are lacking [121]. There are recommended guidelines for animals entering into the hospital environment (service animals, therapy animals, personal pet visitations) [119, 121], but the evidence of the recommended protocols’ effectiveness is based largely on extrapolation from human data and many recommended interventions have not been validated in animals. Numerous documents on the control of MRSA in people have been published [11, 17], and many of the principles may be applied to control in animals. However, caution should be exercised in extrapolating guidelines for MDRO control in people to animals because there may be significant differences in disease epidemiology [118, 163]. Because of their unique microorganism ecosystems and their role as an interactive fomite – a living moveable system independently interacting with individuals and its environment – controls focused on inanimate environmental surfaces may not be effective for animals that enter the hospital or such strategies may result in unintended effects.

While antimicrobial stewardship in human medicine has been shown to decrease HAI prevalence in patients [164], in a four-year study across Australia, the level of antimicrobial exposure in dogs and cats was less than half that for human exposure, and critically-important antimicrobials accounted for only 8% of all the antimicrobials prescribed over the study period [165], so improvement of judicious use of antimicrobials in companion animals may not yield many benefits in some settings. At present, no controlled studies have been conducted to provide data on key questions such as transmission between animals and humans in the hospital, and efficacy of decolonization procedures in animals. Further research is needed in interventions within this One Health domain. For future studies that adopt a One Health approach to evaluate transmission pathways to patients that involve consideration of human, animal, and environmental reservoirs, relevant checklists for study conduct and reporting exist [166, 167].

## Discussion

In this review, we have used a One Health framework to discuss the importance of addressing the hospital environment, the individuals who are treated, work, and visit the hospital, and the animals that directly and indirectly contribute microbial ecosystems, in the prevention and control of hospital-associated pathogens. Hospitals are located within human and animal communities, and the microbial ecosystems of the hospital can be influenced by community-level factors, from individuals who enter the hospital that serve as vectors in the spread of microbes, including pathogens, between the hospital and community. Animals who enter the hospital can also serve in this role and may have altered vector function based on their unique microbial composition, which will be different based on the role they serve (service animals vs. therapy animal vs. personal pet). Antimicrobial pressure in hospitals can be an incubator for MDRO; the cyclic loop between the hospital and community then will continue to foster resistant microbial ecosystems over time.

We have examined current interventions targeted at the hospital environment and to the patients and HCW in the hospital, and the efficacy and drawbacks of each. It has been shown that the most effective intervention programs are multi-modal and designed to minimize individual pathogen exposure before such exposure progresses to colonization and infection. However, environmental decontamination and human hygiene practices decrease but do not eliminate the risk of colonization in other individuals and HAI rates seen in the hospital. A One Health approach may assist in the development of novel research and multi-modal intervention approaches by considering the relationship between the patient, the HCWs, and the hospital environment, and the role of the community. This includes known community-level risk factors for MRSA colonization in patients, such as pet ownership or living in an animal agriculture community [93, 100, 109, 112].

The largest knowledge gap this review exposed was the lack of data within the animal One Health domain. Little research has been done to explore pathogen transmission between animals and humans, within a home or hospital setting, and no studies have looked at the role of decontamination of the animal sector to see if this minimizes bacterial burden on the animal and has downstream effects on reduced transmission to individuals in contact. Compounding this is the need to understand microbial ecosystem dynamics in the context of hospital spread, particularly as such dynamics relate to microbial ecosystems unique to animals or humans, and how such ecosystems may even provide protection against the acquisition of pathogens through the sharing of potentially “beneficial” commensal microorganisms [168–170].

## Conclusions

The complexities of hospital infection control deserve the joint focus of various disciplines. An integrated approach is needed to guide both research pathways and public policy mediations. Utilizing a One Health framework in this brief review allowed us to visualize key gaps in the current knowledge base surrounding hospital infection control and can help direct future research and implementation efforts by suggesting opportunities for advancement in non-traditional conduits.

## Data Availability

Data sharing not applicable to this article as no datasets were generated or analyzed during the current study.

## Abbreviations

HAI: Hospital-associated infections
MDRO: Multi-drug resistance (micro)organisms
MRSA: Methicillin-resistant *Staphylococcus aureus*
CA- MRSA: Community-associated MRSA
HA-MRSA: Hospital-associated MRSA
VRE: Vancomycin-resistant *Enterococcus*
SCCmec: Staphylococcal Cassette Chromosome *mec*
HCW: Healthcare worker
